# Forensic facial reconstruction: A computer tomography study of facial soft tissue thickness in Nigerian adult male multi-ethnic population

**DOI:** 10.1007/s00414-025-03455-9

**Published:** 2025-03-11

**Authors:** Nurudeen Adegbite, Manuela Mura, Haliru Shafiu, Christopher Avery, Waqar Ahmed

**Affiliations:** 1 School of Engineering and Physical Sciences, University of Lincoln,Brayford Pool Campus Lincoln, LN6 7TS Lincoln, UK; 2 Usmanu Danfodiyo University and Teaching Hospital, Garba Nadama Road, Sokoto, Nigeria; 3 Department of Oral and Maxillofacial Surgery, Leicester Royal Infirmary, LE1 5WW Leicester, UK

**Keywords:** Facial soft tissue thickness, Forensic facial reconstruction, Hausa adult male, Human identification, CT reconstruction

## Abstract

Facial soft tissue thickness (FSTT) was measured from computer tomography scans of 55 Nigeria adult males. Forensic facial reconstruction (FFR) with own population FSTT values can be vital in recognition of skeletal remains and has been used as an adjunct in forensic science.There are no published FSTT values for this population. Measurements were obtained at 12 mid-sagittal and 19 bilateral points totalling 50 and by use of a software package called RadiAnt. In comparison to previous studies in Africa, measurements were taken from more points and with a diverse age of 18 to 100 years. Mean FSTT values were determined for these combined Nigerian male ethnicities. These values will suffice in FFR for this population and for the Hausa adult male. This population homogenously showed more soft tissue volumes on the left than the right side at all FSTT points with the most relative difference at the frontal eminence and the least at the mid-point of the masseter muscle. The low relative difference at the mid-masseters may relate with the soft floury diets of this population. These combined Nigerian male ethnicities midline FSTT values showed significant differences in the lower third of the face when compared with other population data. The values for the right side of the face shows even more substantial differences at multiple points when compared with published data of other populations. The change with age compares well with other studies. These data will be applicable for FFR in the CNME than data of other populations.

## Introduction

Forensic facial reconstruction (FFR) is an artistic science which involves reconstructing the face from the skull to allow for recognition in both general forensics and archaeological sciences. FFR is often variously referred to as ’facial approximation’, ‘facial reproduction’, ‘facial reconstruction’, ‘facial restoration’ and ‘facial recreation’. The underlying principle of FFR is the ability to reconstruct the contours of the face by using preferably own population specific facial soft tissue thickness (FSTT) values at pre-determined anatomical points with the skull in the standardised Frankfurt position [1–8]. This modelling of the face from the skull is used to reproduce the antemortem appearance of an individual [6, 7, 9–13]. More commonly, antemortem medical and dental records, deoxyribonucleic acid (DNA) and other forensic science techniques are used for identification [14, 15]. However, for mutilated or decomposed human remains, FFR is a valuable adjunct in identification [4, 16, 17] especially in cases of mass disasters or when minimal individual identifying information is available for example foreigners or when it is impossible to obtain DNA sample or where DNA database is not robust. FFR is based on the premise that because we all have different facial appearances this implies that our skulls are also unique. This uniqueness of the skull-face interface is due to the multiple twenty-two bones which make up the skull [7]. It is much more pertinent because the soft tissues of the whole body will deteriorate making identification difficult if not impossible, the skull will, however, survive the harshest weather. The skull can be use for determination of age, sex and race [7] and with significant accuracy of 98% when used with other skeletons. The Scientific Working Group for Forensic Anthropology (SWGANTH) defined the role of FFR; “to estimate the antemortem facial appearance of an individual from unknown skeletal remains; to suggest the identity of persons to whom the remains might belong; and to capture public attention regarding the case” [13]. In archaeology FFR has been used to rebuild faces of skeletal remains allowing for identification of prominent historical individuals [7, 18–22] for example, Dante Alighieri, Johann Sebastian Bach, King Richard the III, Christian Saint-Nicolosa Bursa. Numerous studies have reported that having an FSTT unique for a population will improve recognition. FSTT data has been documented for different populations; American blacks [16], Uzbek [10], Chinese [23, 24], Colombian [25], Turkish [5, 26], Portuguese [27], French [28], Northwest Indians [4], Japanese [29], American Caucasoids [30], Brazilians [31]. This hypothesis has been buttressed by other studies which have suggested that facial recognition is primarily related to race and sex [32]. Different numbers of FSTT points have been used in previous studies, from as little as 9 in a study by Welcker [7] to 52 [11] points, but as high as 60 in Dent et al. study on Chinese [23]. FSTT has previously been measured manually from embalmed cadavers but, these were shown to be inaccurate due to rapid drying and the embalming process [33]. Whilst cadavers deceased less than 12 h has been shown to give more accurate results [7, 33], most recent FSTT studies have used modern imaging scans; these includes plane cephalometric x ray [34–37], computer tomography (CT) [38–40], magnetic resonant imaging (MRI) [24, 26, 41], cone beam computer tomography (CBCT) [25, 31, 42] and ultrasound scan [11, 34, 43, 44]. CT scan has been shown to be sensitive at demonstrating both hard and soft tissues. CT and MRI are the best techniques for FSTT estimation [45–47]. Though recruiting subjects for CT scans can be difficult due to the associated risk of radiation exposure [45].

Literature search revealed that seven FSTT studies have been published in African male communities as summarised in Table 1 below.

**Table 1:** Summary of FSTT study in Africa

Study name/ year (Abbreviation)	Population studied	M/F	Image type/ number of FSTT points	Number of patients, age range (years)
Aulsebrook et al., 1995 (A°) [34]	Zulus (South Africa)	M	Lateral and oblique cephalometric + ultrasound scan / 54	55, 20 to 35
Phillip and Smith, 1996 (P & S) [46]	Mixed population, White and Black (Cape areas, South Africa)	M/F	Computer tomography scan at 5 mm slides whilst using about 20 slides per patient / 32	32 (16 male, 16 female), 12 to 71
Cavanagh and Steyn, 2011 (C & S) [40]	Black Population (Guateng region, South Africa)	F	Computer tomography thickness of slides not stated. Measurements was from 28 FSTT points midline, 10 of which were midline / 28	154 CT scans, 18 to 35
Hamid and Abuaffan, 2016 (H & A) [37]	Sudanese ethnic Group	M/F	Plane Cephalogram / 20	233, 18 to 35
El-Mehallawi and Soliman [43]	Egyptians	M/F	Ultrasound / 17	204, 20 – 35
Shehata et al., 2023 [48]	Egyptians	M/F	Cone-beam computer tomography / 18 (10 midline, 8 bilaterally)	20 (M = 10, F = 10) / 17—33
This study	6 ethnic groups in Nigeria	M	Computer tomography / 50	55, 18 to 100

These includes studies on Zulu male [34], South African black female [40], South African male mixed race [46] and on Sudanese adults [37]. Other studies in African are the ultrasound scan studies by El-Mehallawi and Soliman [43] and most recently the Shehata et al., [48] both on Egyptians. Also, the Von Eggeling 1904 cadaveric needle puncture study in Namibia [7]. These studies (Table 1) were based on specific populations, mostly limited age range, or limited number of FSTT points, some on single sex, or a limited number of subjects. Many studies have defined FSTTs based on the indices of age [5, 17, 24, 40, 50], sex [17, 24, 28, 39, 41], BMI [28, 41], weight [49] and population studied [5, 17, 26, 27, 42, 50, 52–54] or type of dental malocclusion [23, 37]. Stephan and Simpson [55] had also published an online database which combines most of the available populations data.

The International Committee of the Red Cross (ICRC) registered an increasing number of people missing in African over the last few years. In 2020, 23,000 Nigerians were recorded as missing representing 52.3% of the total missing in Africa [56]. This has been predominantly from northern Nigeria. The number of people missing had increased by 75% in August 2024 when compared to figures from 2019, Table 2. These people may never be found alive, and many may be mutilated or decomposed. Having own population specific FSTT values may help improve FFR and enhance identification. There is no published study of FSTT specifically for Nigerian populations.

**Table 2:** Number of missing people in Africa

Year	Missing persons
2019	40,708
2020	44,000
2024	71,000

The main aim of this study is to establish the FSTT values for adult males in a Nigerian multiethnic community by use of CT images. To determine the impacts of age, sex, and body mass index (BMI) and compare these FSTT measurements with other existing database of African origin and some other population FSTT data.

## Method and materials

This study was based on analysis of Computer Tomography (CT) scans. Data including Computer Tomography (CT) scans of the head and neck region were collected on live subjects over a 9-month period from March to November 2023 at the Uthman Dan Fodio University in Sokoto, Nigeria. A total of 65 subjects were recruited. The age ranged from 18 to 100 years.

### Ethics approval

Ethical approval was granted by the University of Lincoln research ethics committee and all subjects’ received data and scans were completely anonymised in line with the principles stated by the Nigeria Data Protection Regulations (NDPR) for public institutions. We complied strictly with the ethical principles for medical research involving human data as stated by the World Medical Association in the October 2013 Principles of Helsinki [51].

### Computer tomography (CT) imaging

The CT scanner used for data collection in this study was a 16 slice helical machine manufactured by Hangwei Medical Systems Co. Ltd. It’s a revolution Apex CT by General Electric (GE). Axial, sagittal, and coronal CT views were collected for all subjects. It produced images of 2.5 mm thickness. To avoid risk of radiation these were CT scans done for reasons unrelated to this study. Each scan was selected based on the exclusion criteria stated below. A shared online site was created, images were remotely downloaded for analysis.

### Population studied

Data was collected from the 3 major ethnic groups in Nigeria, the Hausa, Igbo and Yoruba tribes. This northwestern part of the country is primarily home to the Hausas. The northern part of the country accounts for a greater number of people missing in Nigeria. During data collection we identified a total of six ethnic groups, these included; the Hausa (39 subjects), the Fulani (7), Igbo (2), Yoruba (2), Nupe (1) and Dakarkari (4).

### Data collection

A reproducible form (Form A) was used for data collection for each patient. Data collected for each subject included a unique number, name of hospital, date of birth, sex at birth, body mass index (BMI), ethnicity, date of imaging. Anonymity of the patients were ensured by using individual identifier numbers. In order to be consistent with other studies exclusion criteria included histories of head and neck trauma, facial swelling, orthodontic treatment / orthognathic surgery, bony abnormality, cleft lip and palate [4, 5, 40]. In addition, subjects with tribal marks were excluded as this is a common cultural practice [57] and also those with history of inter-tribal marriages over 2 generations at least. An administrator of indigenous origin was recruited for biodata collection. A maxillofacial radiologist at this hospital collected and selected appropriate CT scans for analysis. Interactive online trainings were undertaken before data collection.

### Body mass index (BMI) characterisation

BMI was calculated using the standard formula BMI = weight (kg) / height (metres square). It was noted that different BMI classifications had been used in previous FSTT studies. A literature search revealed 3 different definitions of underweight, normal and overweight as; < 20, 20–25, > 25 [5, 28]; < 18.5, 18.5–23.9, > 23.9 [23]; < 18.5, 18.6–24.9, 25.0– 29.9 [25]. In this study we adopted the WHO classification; for 18 years and above: < 18.5 (underweight), > 18.5 – 24.9 (normal weight), > 25–29.9 (pre-obese) and > 30 (obese).

### Image post-processing for landmark measurements

The RadiAnt software package was used in estimating all FSTT values, Medixant Company, PACS Dicom Viewer, version 2023.1 (64-bit). This software has the ability to open and demonstrate different scans including MRI, CT, USS, PET (Positron Emission Tomography). The software displays both the soft and hard tissue windows and it supports all types of DICOM (Digital Imaging and Communications in Medicine) images including monochromatic (e.g. chest x-ray (CXR), CT) and colour (e.g. 3D reconstructions) and static images (e.g. CXR, CT). This software has a local archive for images to be stored and accessed when needed. It is able to process many gigabytes of data, the 64-bit version can handle images of more than 4 GB and an installer size of 7 MB. It is able to fast reconstruct images in multiple orthogonal planes (sagittal, axial, coronal, oblique). The software automatically harmonised series of images within the same plane (e.g. CT images of pre and post contrast medium). The 3D VR (volume rendering) tool allows checking for any soft or hard tissue abnormality including scars and tribal mark (traditional facial identification marks), cleft lip and facial deformity. The software scalpel allows for unwanted areas to be removed. The brightness, contrast, negative modes allows identification of foreign bodies that could cause soft tissue swelling or displacement. There is a preset window settings for computer tomography. The angle measuring tool allows for FSTT to be measured at 90° degree from bone to skin surface. The pen tool allows for sketching and marking of FSTT points. The multi-touch feature is suited for Windows 8 or Windows 10 and translates into more than 20 languages. Numerous sequences of same study or various studies can be opened concurrently in the same or multiple windows for comparison purposes. The variety of tools allows for images to be rotated to 90°, 180° and manipulated as desired. This was accessed at www.radiantviewer.com.

In this study FSTT measurements were taken at 12 midlines and 19 bilateral defined points making a total of 50 expected for each subjects. These FSTT points were mostly replica of points as stated in previous studies by Sahni et al., [9], Bulut et al., [5], Wilkinson [7], Auslebrook et al., [34] and De Greef et al., [38, 44], Table 3.

**Table 3:** Definitions of FSTT points

	Facial soft tissue points (FSTT)	Definitions of FSTT points	Commonly used synonyms
Midline			
1	Vertex (v)	The highest point on the head	
2	Supraglabella (sg)	The most anterior midline points on the forehead	Ophryon (of)
3	Glabella (g)	Most prominent point between supra-orbital ridges in midsagittal plane	
4	Nasion (n)	Midpoint of the suture between the frontal and the two nasal bones	
5	Mid-Nasal (ns)	The midpoint of the nasal bone, which is between the nasion and rhinion	
6	Rhinion (rh)	The end of the nasal bones at the cartilage-bone junction	End of the nasal
7	Midphiltrum (mp)	Midpoint of the philtral column- located by the length from the midline and midway point between prosthion to the subspinale. Measured from the deepest point (point A) of the maxillary alveolar bone	
8	Upper lip border (ls)	The most anterior midpoint of the upper vermilion line measured from the prosthion Measured from between the central incisors at the level of the cementum–enamel junction to the vermillion border The prosthion is midpoint between the upper central incisors at the level of the cementum–enamel junction	Supradentale or alveolare or Labiale superius
9	Lower lip border (li)	The midpoint of the lower vermilion line Measured from between the lower central incisors at the level of the cementum–enamel junction to the most anterior of the vermillion of lower lip	Infradentale or Labiale inferius
10	Labiomental (lm)	The midpoint of the labiomental grove. The deepest midline point furrow on the mandible between the teeth and the chin prominence	Chin-lip fold sublabiale or supramentale
11	Mental eminence (me)	The most anterior midpoint of the chin	Pogonion
12	Menton (mn)	The lowest medial landmark beneath the chin	Gnathion or Beneath chin
Bilateral			
13	Frontal eminence (fe)	Centred on pupils most anterior projections at both sides of the forehead A point on the projections at both sides of the forehead (centred on the pupils / lies above the midpoint of the eyebrow). It is located as the most protruding point of the frontal bone along the axis of a line (drawn from ecthocanthion to endocanthion) that vertically bisects the orbit	
14	Supraorbital (os)	Above the orbit, it’s the highest point of the upper margin of the orbit (centred on the pupil). Located from a point mid-way between the ecthocanthion to endocanthion and by drawing a perpendicular line up to the supraorbital margin	
15	Infraorbital (oi)	The lowest point on the lower margin of the orbit. Located from a point mid-way between the ecthocanthion to endocanthion and by drawing a perpendicular line up to the Infraorbital margin	Orbitale (or) suborbital
16	Supraglenoid (sg)	This is the root of the zygomatic arch. It is anterior and superior to the external auditory meatus	
17	Zygomatic arch (zy)	Most lateral point of the zygomatic arch	Zygion
18	Lateral Orbit (lo)	This is an anterolateral point on the zygomatic bone. It is the lowest point on the suture between the maxillary and zygomatic bones. It is lined up vertically to the lateral orbital margin on the zygomatic bone	
19	Lateral Glabella (lg)	A point on the medial orbit at the junction of the frontal, maxillary, and lacrimal bones	
20	Lateral nasal(ln)	A point on the side of the nose in line with the endocanthion. It is a point on the Frankfurt horizontal plane at the side of the bridge of the nose	
21	Inferior malar (Im)	Most protruding point of the maxilla, lies beneath the zygomatic process at a point on a vertical line with the infraorbital and supraorbital points. It is centred on the vertical line from pupil, and just inferior to zygomatic process	Lateral maxilla
22	Lateral nostril (lno)	This is a point just next to the most lateral point of the alar border	
23	Supra-canine (sc)	This is a point on the superior alveolar ridge of the upper canine. It is on the horizontal level of the mid-philtrum and lines up vertically with the cheilion. It is measured from the maximum bulge of the upper canine prominence	
24	Infra-canine (ic)	This is a point on inferior alveolar ridge of the lower canine. It is vertically lined up with the cheilion, on the horizontal level of the chin–lip fold	Subcanine
25	Mid-lateral orbit (mlo)	This is a point just next to the lateral orbital border	
26	Supra-M2 (sm2)	This is a point on the superior alveolar ridge of the second upper molar	
27	Infra-M2 (im2)	This is a point on the inferior alveolar ridge of the second lower molar	
28	Mid-masseter (mm)	A point located halfway between the supraglenoid and the gonion, located about the middle of the masseter, (*located from coronal view*)	
29	Gonion (go)	The most lateral point at the angle of mandible (located on the angle of the mandible),	
30	Occlusal line (ol)	A point on the anterior part of the ascending ramus in alignment with the line where the teeth occlude anterior lateral?	
31	Mid-mandible (mdm)	A point on the inferior border of the mandible, vertically lined with the supra-M2, (Bulut et al., 2018) *(measured from coronal view)*	

**Fig. 1:**
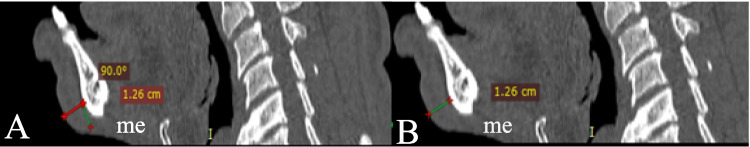
Measuring of FSTT (**A**) showing angle placement (**B**) final measurement

However, contrary to other studies the inferior malar was labelled as the lateral maxilla, this better describes its lateral position. Each image was easily downloaded into RadiAnt and desired FSTT points were located on its relevant orthoslice. Midline FSTT were measured on the sagittal view using the nasal septum and the dentition as a guide. Bilateral FSTT points were measured from the axial view except for the mid-masseter point which was recorded from the coronal view by locating the origin and insertion of the masseter muscle and estimating the mid point between these and measuring its thickness at this point from the bone to skin. Bilateral measurements were more time consuming to perform; whilst midline FSTT were obtained from one orthoslice, bilateral FSTT involved identifying each FSTT mostly on different orthoslice. Measurement at each FSTT point was taken at 90° from bone to the skin surface with the skull in a Frankfurt position, this is similar to that described in previous studies [7, 34, 40, 58, 59]. For example, Fig. 1(A) illustrates placement of angle tool at 90° at the mental eminence. The angle tool placing at 90° was time consuming as it could not be automated.

**Fig. 2:**
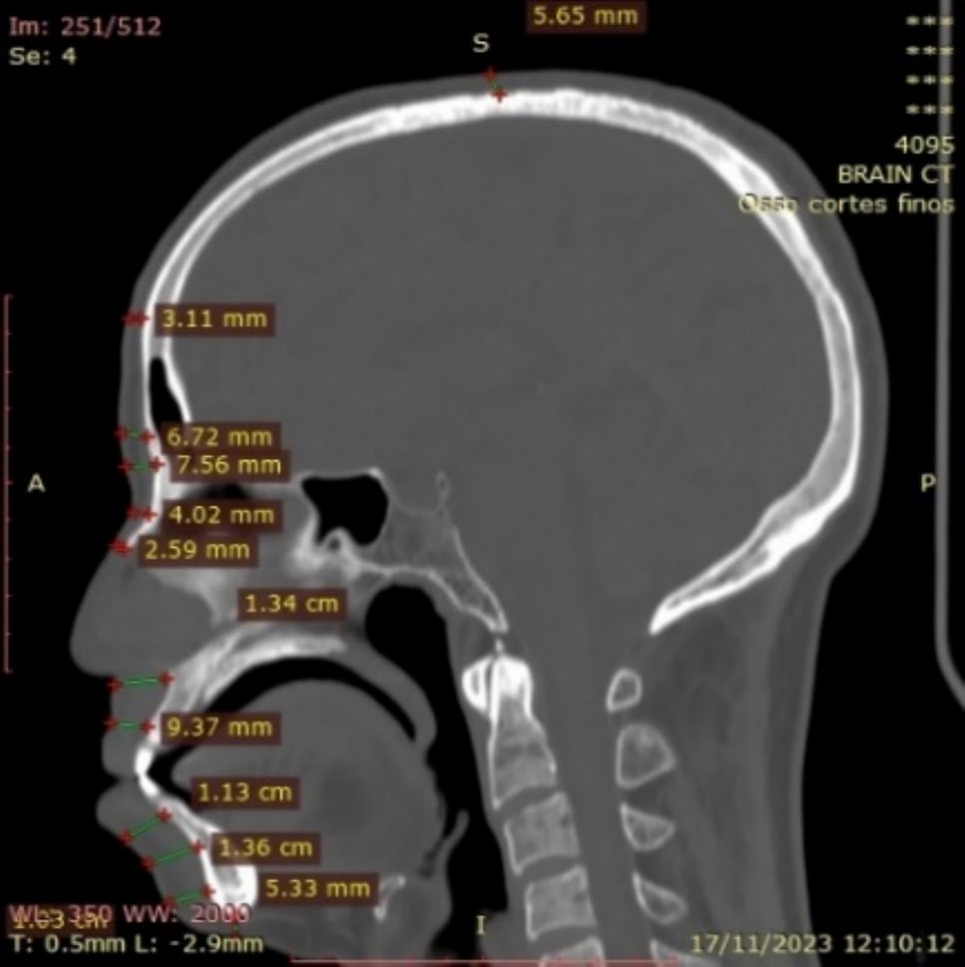
Mid-line FSTT measurements

Secondarily after placing at 90° at the identified FSTT point a measuring tool was then placed over this linear line to estimate the representative distance from bone to skin surface as illustrated above, Fig. 1(B). The angle tool could then be removed leaving only the estimated and the linear measurement line. The software pen tool allowed for labelling of the processed images at FSTT points if needed. Figure 2 is an example of estimated midline FSTT points.

**Fig. 3:**
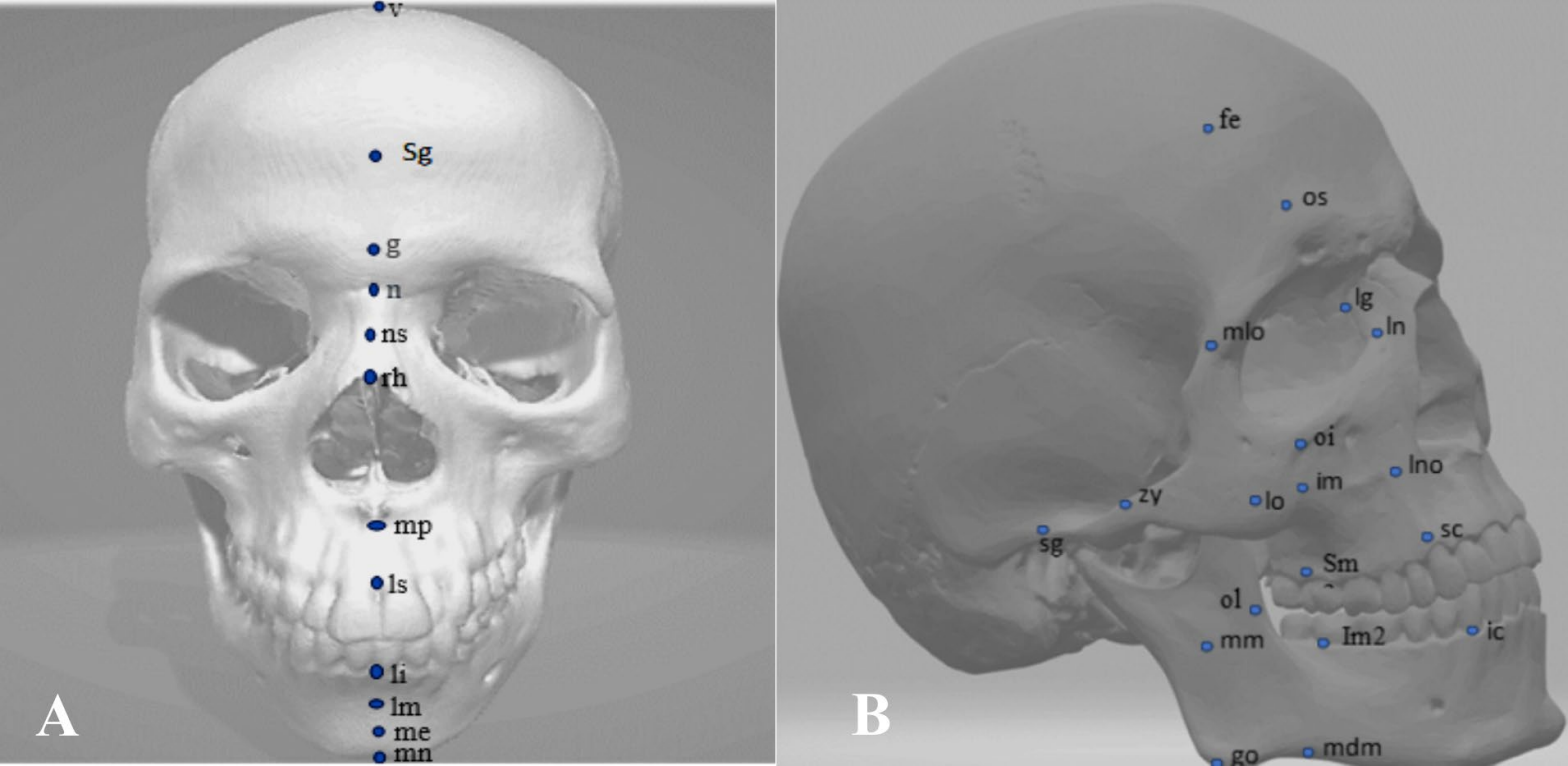
**A** 12 midline (**B**) 19 bilateral FSTT points (right side)

Images could then be exported as JPEG, videos into word documents. Figures 6 and 7 (Section "Appendix") illustrates some measured FSTT points whilst Fig. 3 illustrates these points on the skull.

**Fig. 4:**
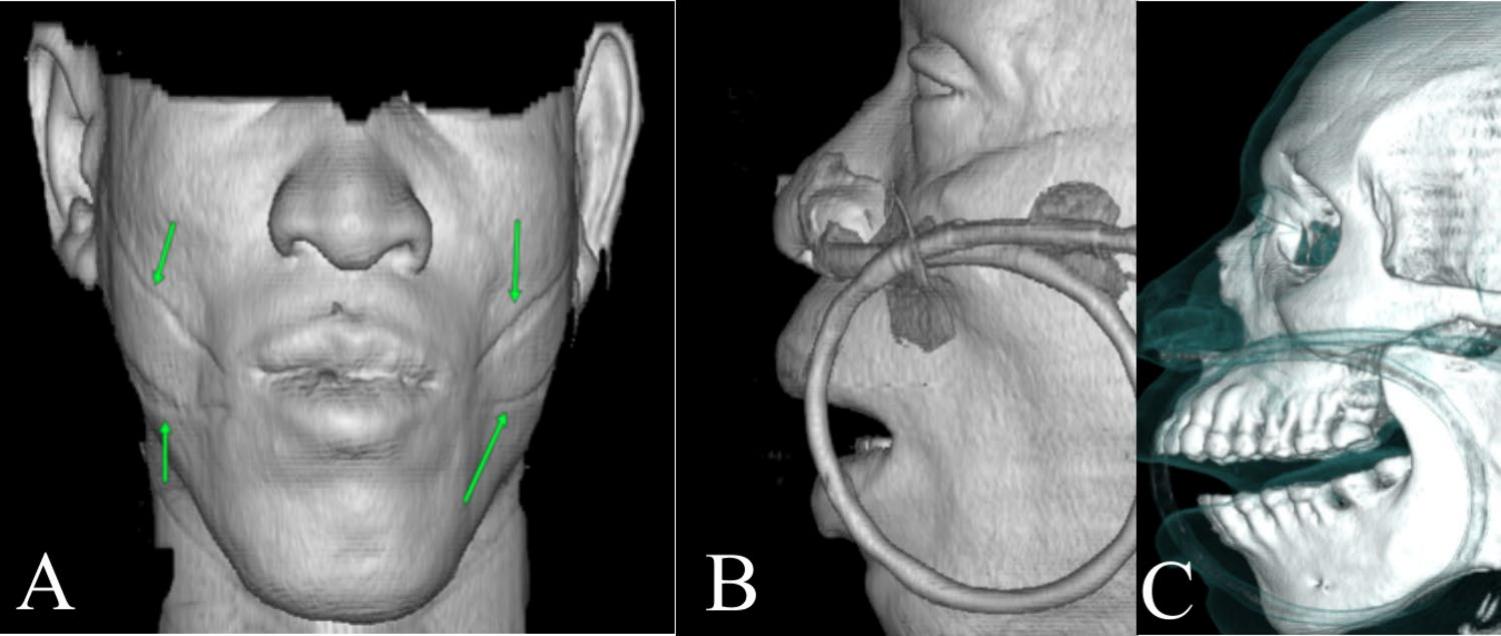
A subject with tribal mark denoted by green arrows. (**A**) Facial tribal marks denoted by green arrows (**B**) Naso-gastric tube (**C**) Naso-gastric tube in a CT bony window also showing soft tissues

65 CT scans were analysed, using the 3D reformat tool of the software, 10 scans were discarded either because of presence of gauze, ethnic specific tribal marks as illustrated in Fig. 4A, naso-gastric tube distorting the soft tissue Fig. 4B and C, distorted images or artifacts, evidence of patient movement or the scans not demonstrating significant areas of the face. A total of 4003 manual measurements were done and analysed.

#### Statistical analysis

Statistical analysis of these measurements were performed using SPSS (IBM Corporation, 1989, 2023). In order to minimise intra- and inter-observer errors the FSTT measurements of the first ten patients were done thrice by the same observer. These initial measurements were done to check for errors of normality and enhance proficiency in measurements. The statistical p-values were consistent with a proficient level of accuracy in measurements of < 0.05. The same observer analysed the rest of the scans.

## Results

All patients were classified into age brackets, Table 4. The Hausas are the majority because this region is home largely to them.

**Table 4:** Definitions of FSTT points

Age range	Dakarkari	Fulani	Hausa	Igbo	Nupe	Yoruba	Total count
(a) 18–32	1	1	12	–	1	2	17
(b) 33–47	2	1	8	–	–	–	11
(c) 48–62	1	3	13	2	–	–	19
(d) 63–77	–	2	3	–	–	–	5
(e) 78–92	–	–	2	–	–	–	2
(f) 93–100	–	–	1	–	–	–	1
Grand Total	4	7	39	2	1	2	55

The most represented age brackets were the (a) 18–32 years and (c) 48–62 years. The least populated was (f) with only one patient aged 100 years. This is probably a reflection of the reducing life span of this population of 54.3 years for males [65]. Not all patients had BMI checked as some pre-existing CT scans were added. Of these 55 males, 27 had BMI recorded and 28 had no BMI recorded. The number of patients with recorded BMI of each population are illustrated in Table 5.

**Table 5:** Number of males with BMI by ethnic group

Subjects with recorded BMI by ethnicity	
Hausa	19
Fulani	2
Igbo	2
Yoruba	1
Nupe	0
Dakarkari	3
Total with BMI	27

Most literatures suggest that FSTT values increases with higher weights and BMI [7, 49, 50, 52]. Descriptive statistics of mean, minimum (min), maximum (max), range, standard deviation (SD) and the number of all measured points were calculated for the CNME in Tables 6 and 7 below. If male but age, not known these values will suffice as the average FSTT values for reconstruction of a Nigerian adult male of 18 years and above. Such average values with no age classification has been used in many FSTT studies [40, 42]. The midline FSTT with the lowest value was the rhinion, this is similar with other studies [5, 7, 40, 42, 53]. The highest value was at the midphiltrum, then the labiomental, this is similar to the 19–34 black American values, Manhein et al. [53] and other published works [5, 25].

**Table 6:** Average values for CNME males FSTT midline points (mm)

FSTT point	Mean	Min	Max	Range	SD	Count
Vertex	6.39	2.49	9.53	7.04	2.01	53
Supraglabella	4.56	2.92	13.5	10.58	1.77	54
Glabella	5.46	3.48	12.1	8.62	1.53	54
Nasion	6.09	3.04	11.6	8.56	1.75	55
Mid-Nasal	3.97	1.9	7.57	5.67	1.24	55
Rhinion	3.35	1.77	9.72	7.95	1.15	55
Midphiltrum	12.64	7.75	16.8	9.05	2.60	54
Upper lip border	10.17	7.15	13.8	6.65	2.04	54
Lower lip border	10.79	8.07	16.8	8.73	1.84	55
Labiomental	12.40	9.55	17.1	7.55	1.52	55
Mental eminence	10.36	6.19	15.3	9.11	2.55	54
Menton	6.29	2.99	10.2	7.21	2.13	52

**Table 7:** Average values for males FSTT right and left sides (mm)

FSTT points	Right						Left						*D of lt & rt		Hwang et al
	Mean	Min	Max	Range	SD	Count	Mean	Min	Max	Range	SD	Count	lt – rt	lt > rt (%)	rt > lt %
Frontal eminence	4.77	2.77	8.44	5.67	1.42	53	5.16	2.98	14.5	11.52	1.85	54	0.39	8.18	2.0
Supraorbital	5.73	2.33	10.2	7.87	1.78	53	6.05	2.45	10.8	8.35	1.79	53	0.32	5.58	2.0
Infraorbital	9.44	4.68	15.6	10.92	3.30	51	9.59	4.53	15.5	10.97	3.38	51	0.15	1.59	−1.2
Supraglenoid	12.55	6.47	20.2	13.73	3.60	54	13.48	7.08	21.7	14.62	3.67	54	0.93	7.41	−0.6
Zygomatic arch	6.26	3.34	10.9	7.56	2.16	54	6.56	3.17	11.3	8.13	2.17	54	0.3	4.79	−2.1
Lateral Orbit	6.06	3.55	11.1	7.55	1.86	54	6.38	3.24	10.4	7.16	1.93	54	0.32	5.28	−0.8
Lateral Glabella	4.92	2.51	10.4	7.89	1.67	54	5.05	2.58	11.8	9.22	1.84	54	0.13	2.64	−1.6
Lateral nasal	7.43	3.96	13.2	9.24	2.35	52	7.75	3.91	13.1	9.19	2.5	52	0.32	4.31	1.8
lateral maxilla	20.09	7.87	29.2	21.33	5.90	52	21.02	4.77	29.7	24.93	6.00	53	0.93	4.63	0.3
Lateral nostril	9.33	7.13	12.9	5.77	2.23	53	9.83	7.16	17.3	10.14	2.85	52	0.5	5.36	1.4
Supracanine	8.80	5.95	12.3	6.35	2.73	51	9.11	5.95	12.3	6.35	2.77	51	0.31	3.52	0.3
Infra-canine	8.42	6.00	12.3	6.3	2.49	51	8.81	6.01	12.2	6.19	2.64	51	0.39	4.63	1.5
Mid-lateral orbit	3.61	1.77	5.57	3.8	1.15	52	3.8	2.07	5.67	3.6	1.11	53	0.19	5.26	0.9
Supra-M2	19.93	10.7	37.6	26.9	5.46	54	20.74	2.69	37.0	34.31	6.31	54	0.81	4.06	−0.6
Infra-M2	17.34	10.1	26.1	16.00	4.11	54	17.96	10.9	26.2	15.3	4.28	54	0.62	3.58	−0.5
Mid-masseter	21.54	7.08	31.5	24.42	6.31	52	21.87	7.01	31.8	24.79	6.57	52	0.33	1.53	−2.6
Gonion	16.57	9.02	33.2	24.18	6.09	52	17.61	9.86	32.5	22.64	6.3	52	1.04	6.28	−3.7
Occlusal line	19.51	9.55	34.3	24.75	6.49	52	20.6	9.37	32.1	22.73	6.63	52	1.09	5.59	−0.2
Mid-mandible	11.71	5.61	26.9	21.29	5.63	47	12.29	6.19	24.3	18.11	5.72	47	0.58	4.95	−3.1

*D = relative difference

Bilateral measurements (right and left) were analysed separately in Table 7. The differences (*D) between the right and left sides revealed that the CNME homogeneously had minutely more soft tissues on the left side of face at all FSTT points as compared to the right side. Previous study by Sahni et al., [4] on Indian adults, had also reported an homogeneous but minutely increased FSTT values on the left than the right side of the face. The FSTT point with the least relative difference (RD) was the mid-masseter (1.53%). This may be because the Hausas and this population thrives on a predominantly soft diet of grains including maize, millet, rice, these are very often grounded into flours to be processed into a variety of dishes known as tuwo in the Hausa dialect [57]. The other dietary intakes are equally soft and includes Kosai (ground beans), funkaso (wheat flour), konu / koko (porridge with sugar), spinach and except for suya which is a sheep or cow meat spiced and skewered. This may also relate with the dental hygiene, teeth loss or edentulous dentition [66]. The most varied RD was the frontal eminence (8.18%). The left side of the face had on average 4.69% more soft tissues when compared with the right. In a similar study on Korean adults, Hwang et al., [42] revealed varied differences between the right and left side of the face, with the differences not as homogeneously one-sided as compared to this study, Table 7. Hwang et al., reported highest RD was at the gonion and the lowest was at the occlusal line.

Furthermore, average FSTT values by age brackets for the CNME were estimated as presented in Tables 8 and 9. This type of descriptive analysis has been published in other studies [5, 16, 17, 38, 40, 53]. These values will be applicable for a Nigerian male adult of any ethnicity and of known age estimate. The lowest FSTT for all age brackets was at the rh and mlo, this is like other published studies [5, 25, 53]. The highest value was consistently at the mm. The mm increased in early life but reduces in thickness from the 48–62 age bracket.

**Table 8:** Average values for CNME FSTT points by age brackets (mm)

FSTT points	18–32 (A)						33–47 (B)						48–62 (C )					
	Mean	Min	Max	Range	SD	Count	Mean	Min	Max	Range	SD	Count	Mean	Min	Max	Range	SD	Count
Vertex	7.27	2	9.53	7.53	2.97	15	7.11	5.35	8.9	3.55	1.23	11	5.89	5	6.68	1.68	0.49	19
Supraglabella	4.85	3.04	13.5	10.46	2.57	16	4.50	2.92	7.02	4.1	1.62	11	4.57	3.11	8.02	4.91	1.24	19
Glabella	6.06	4.04	12.1	7.56	2.25	16	5.58	3.81	8.31	4.5	1.43	11	5.26	4.1	6.72	2.62	0.77	19
Nasion	6.63	4.73	11.6	6.87	1.80	17	6.10	3.04	11.3	8.26	2.54	11	6.12	3.1	8.2	5.1	1.32	19
Mid-Nasal	4.37	2.72	7.57	4.85	1.37	17	4.02	2.37	6.51	4.14	1.48	11	4.00	2.05	5.98	3.93	0.90	19
Rhinion	3.31	2.02	4.62	2.6	0.80	17	3.33	2.26	4.52	2.26	0.67	11	3.64	1.97	9.72	7.75	1.60	19
Midphiltrum	13.31	7.75	16.8	9.05	2.27	17	12.93	11.1	15.5	4.4	1.42	11	12.28	8.73	15.7	6.97	1.98	19
Upper lip border	10.92	7.83	12.7	4.87	1.25	17	10.56	8.29	13.8	5.51	3.58	10	9.95	7.94	12.5	4.56	1.13	19
Lower lip border	10.44	8.23	14.5	6.27	1.68	17	11.04	8.9	14.5	5.6	1.71	11	11.03	8.16	16.8	8.64	2.08	19
Labiomental	11.91	9.55	14.4	4.85	1.31	17	12.28	11	13.7	2.7	0.94	11	12.97	10.2	17.1	6.9	1.92	19
Mental eminence	10.11	6.19	14.8	8.61	1.93	17	10.07	6.29	12.3	6.01	1.85	11	11.09	6.74	15.3	8.56	3.54	18
Menton	6.33	3.82	9.96	6.14	1.78	17	6.19	4.62	9.19	4.57	1.47	11	6.45	2.99	10.2	7.21	2.90	16
Frontal eminence	4.46	2.77	5.65	2.88	1.86	15	4.85	3.47	7.38	3.91	1.09	11	4.95	2.95	8.44	5.49	1.43	19
Supraorbital	6.14	4.27	10.1	5.83	2.49	16	5.59	4.06	6.94	2.88	1.00	11	5.84	2.33	10.2	7.87	2.08	18
Infraorbital	9.87	5.07	15.2	10.13	4.84	14	9.65	6.32	12	5.68	1.86	11	9.15	4.68	15.6	10.92	3.22	18
Supraglenoid	12.58	6.47	20.1	13.63	4.61	17	13.19	9.11	17.1	7.99	2.41	11	12.78	8.89	20.2	11.31	4.48	18
Zygomatic arch	6.27	3.34	10.7	7.36	2.48	17	7.00	4.49	10.3	5.81	2.23	11	6.14	3.74	10.9	7.16	2.35	18
Lateral Orbit	6.01	3.55	11.1	7.55	2.41	17	6.68	4.16	9.62	5.46	1.97	11	6.10	4.04	9.77	5.73	1.92	18
Lateral Glabella	5.50	3.21	9.58	6.37	2.00	17	4.36	2.88	6.67	3.79	1.08	11	5.11	3.11	10.4	7.29	2.03	18
Lateral nasal	8.41	5.28	13.2	7.92	3.74	15	6.98	4.86	9.49	4.63	1.20	11	7.14	3.96	9.91	5.95	2.22	18
lateral maxilla	20.23	12.7	24.9	12.2	7.22	16	20.79	15.6	27.1	11.5	7.65	10	20.45	16.2	29.2	13	5.55	18
Lateral nostril	9.77	7.13	12.8	5.67	3.46	16	9.41	7.21	11.5	4.29	1.22	11	9.40	7.16	12.9	5.74	2.54	18
Supracanine	9.54	7.5	12.3	4.8	3.34	16	9.15	7.15	11.9	4.75	3.08	10	8.50	5.95	11.1	5.15	2.40	18
Infra-canine	8.91	7.98	10	2.02	3.49	15	8.52	6.95	12.3	5.35	2.98	10	8.20	6	10.7	4.7	2.24	18
Mid-lateral orbit	3.94	2.77	5.57	2.8	1.54	16	3.66	2.29	5.5	3.21	1.48	10	3.62	2.27	4.68	2.41	1.02	18
Supra-M2	21.24	16.9	29.1	12.2	5.85	17	20.05	13.6	28.4	14.8	5.16	11	20.61	10.7	37.6	26.9	7.25	18
Infra-M2	18.50	15	24.8	9.8	5.37	17	18.51	12.5	26.1	13.6	4.59	11	16.79	11	20.4	9.4	4.49	18
Mid-masseter	22.49	18.3	27.7	9.4	7.88	16	23.22	20.3	27	6.7	7.41	10	20.88	12.5	31.5	19	6.14	18
Gonion	16.45	9.02	24.8	15.78	6.92	16	17.02	9.17	23.4	14.23	6.91	10	17.45	9.66	33.2	23.54	6.63	18
Occlusal line	20.63	15	27.2	12.2	7.56	16	20.04	10.6	26.9	16.3	7.87	10	19.09	9.55	34.3	24.75	6.94	18
Mid-mandible	11.27	6.25	17.3	11.05	5.41	15	12.03	5.61	16.6	10.99	5.99	9	12.55	6.19	26.9	20.71	6.99	15

**Table 9:** Average values for CNME FSTT points (mm) by age brackets (mm)

FSTT points	63–100 (D)						Differences between		
							18–32 & 63–100	18–32 & 63–100	18–32 & 63–100
	Mean	Min	Max	Range	SD	Count	A-B	A-C	A-D
Vertex	4.00	2.49	5.02	2.53	0.97	8	0.16	1.38	3.27**
Supraglabella	4.11	3.39	4.78	1.39	0.57	8	0.35	0.28	0.74
Glabella	4.67	3.48	5.67	2.19	0.78	8	0.48	0.8	1.39
Nasion	5.32	3.35	6.5	3.15	1.05	8	0.53	0.51	1.31
Mid-Nasal	3.21	1.9	4.36	2.46	0.83	8	0.35	0.37	1.16
Rhinion	3.01	2.06	4.26	2.2	0.85	8	−0.02	−0.33	0.3
Midphiltrum	11.69	9.64	14.2	4.56	4.37	7	0.38	1.03	1.62*
Upper lip border	8.48	7.15	11	3.85	1.44	8	0.36	0.97	2.44*
Lower lip border	11.00	8.86	13.2	4.34	1.53	8	−0.6	−0.59	−0.56
Labiomental	12.39	10.5	13.8	3.3	1.14	8	−0.37	−1.06	−0.48
Mental eminence	10.14	6.27	12.9	6.63	2.20	8	0.04	−0.98	−0.03
Menton	6.11	4.67	8.58	3.91	1.25	8	0.14	−0.12	0.22
Frontal eminence	4.71	2.89	5.85	2.96	0.86	8	−0.39	−0.49	−0.25
Supraorbital	4.92	3.4	6.18	2.78	0.81	8	0.55	0.3	1.22
Infraorbital	8.92	7.01	11	3.99	1.44	8	0.22	0.72	0.95
Supraglenoid	11.23	7.68	16.5	8.82	2.64	8	−0.61	−0.2	1.35
Zygomatic arch	5.63	3.9	8.74	4.84	1.68	8	−0.73	0.13	0.64
Lateral Orbit	5.38	3.93	7.23	3.3	1.09	8	−0.67	−0.09	0.63
Lateral Glabella	4.19	2.51	5.51	3	1.25	8	1.14	0.39	1.31
Lateral nasal	6.85	5.55	8.02	2.47	0.71	8	1.43	1.27	1.56*
lateral maxilla	17.48	7.87	22.6	14.73	4.38	8	−0.56	−0.22	2.75*
Lateral nostril	8.12	7.17	10	2.83	0.90	8	0.36	0.37	1.65*
Supracanine	7.21	6.07	9.4	3.33	2.76	7	0.39	1.04	2.33*
Infra-canine	7.77	6.52	9.61	3.09	0.97	8	0.39	0.71	1.14
Mid-lateral orbit	3.05	1.77	3.89	2.12	0.63	8	0.28	0.32	0.89
Supra-M2	15.30	13.6	18.7	5.1	2.12	8	1.19	0.63	5.94**
Infra-M2	14.64	10.1	17.6	7.5	2.59	8	−0.01	1.71*	3.86**
Mid-masseter	18.39	7.08	24.6	17.52	5.33	8	−0.73	1.61*	4.1**
Gonion	14.08	9.93	21.5	11.57	3.93	8	−0.57	−1	2.37*
Occlusal line	17.03	12	22.6	10.6	3.62	8	0.59	1.54*	3.6**
Mid-mandible	10.14	7.16	16.8	9.64	3.04	8	−0.76	−1.28	1.13

*= indicates significant level, **= more significant

Most CNME FSTT points showed increase in values from the 18–32 to 33–47 then more likely to decrease in the 48–62 and decreased further in the 63–100 as displayed in Table 9. This is similar to FSTT characteristic changes as reported in other studies of age brackets [5, 7, 53]. Such sub-classification often reduces the number of subjects in each age brackets data as illustrated in Table 10. Coskun had 5 subjects for sub-classification of the 35–44, De Greef had 3, 1 and 2 in the 30–39, 40–49 and 50–59 respectively and Manhein had 3 males in the 35–45.

**Table 10:** Number of subjects per age bracket in different FSTT study (mm)

Study name	Total number of subjects studied	Age bracket used (years)	Number of subjects in each age bracket M, F
Coskun [60]	100	18–34 35–44 45–54 55–64 ≥ 65	8, 8 5, 5 9, 6 7, 6 21, 25
De Greef, 18–29 with BMI<20 in male [11]	457 (though total male in this 18–29 regardless of BMI= 211)	18–29 30–39 40–49 50–59	28 3 1 2
Manhein (Blacks of N weight) [53]	66	19–34 35–45 46–55	19, 18 3, 21 0, 5
Manhein (Whites of N weight) [53]	130	19–34 35–45 46 −55 >56	28, 52 10, 15 5, 6 5, 9
Tilotta [64]	85	20–40 41–65	17, 40 9, 19
Moritsugui 2022 [61]	101	18–30 31–40 ≥41	18, 22 13, 14 14, 20
This study	55	18–32 33–47 48–62 63–100	18 11 19 8
This study (based on the Hausas only)	38 (Hausa)	18–32 33–47 48–62	13 7 13

The 63–100 age bracket had less soft tissue thickness than the other age brackets for both midline and bilateral FSTTs except at the lm, li and fe. 12 of these 31 FSTT points showed recognizable changes with age as indicated by * in Table 9. The bilateral FSTT showed changes with age at 9 of 19 points, these were mostly in the posterior mandible at the sm2, mm, im2, ol, lmx, go, sc, ln and lno. For the midline FSTT the v, ls and mp showed most changes with age. Not all FSTT showed relationship with age. The least sensitive with age were the mn, rh, li and fe.

## Discussion

Over the last few years there has been exponential increase in the number of people missing in Africa [62, 63]. Wherever needed, FFR will become useful in relocating skeletal remains in these communities. In this study FSTT values were created for adult males of 6 different ethnicities in north-west Nigeria. FSTT was measured at 50 points, 12 midline and 19 bilateral from CT scans. CT scan has been proven to be proficient in demonstrating hard and soft tissues. But the inherent risk of radiation made it difficult to recruit subjects. Measurement of FSTT was by use of RadiAnt software package. The statistical characteristics of mean, maximum, minimum, range and standard deviation of these FSTT values have been calculated for the combined Nigerian ethnicities, also by age brackets. In comparing this value with other data, a benchmark of at least 1.5mm was used recognisable difference because differences of < 1mm has been said to be irrelevant [67].

### Analysis of mid-line data

#### Effects of age

Tables 8 and 9 showed that most FSTT values decreased with age, this is more significant in the 63–100 age bracket. The midline FSTT most sensitive with age changes were the v, ls and mp. However, not all FSTT showed relationship with age, the least sensitive with age were the mn, rh and me, li and lm. FSTT with the least value was the rh, and highest values even by age brackets were at the mp and the lm points. The rh showed insignificant changes due to the minimal thickness of this FSTT, this has been reported in many other publications [5, 7]. Similar to the CNME, Manhein [53] study on black American population showed that there was correlation with age at five of the 19 FSTT points. In the same study, Manhein [53] reported that correlations existed at eight of 19 points for white Americans. The reports of other populations have also shown changes with age changes, these includes published works by His, and also by Czekanowski, both on white European adult males. Whilst Czekanowski [7] showed that all FSTT increased with age but decreased after fifty years, His [7] showed that most FSTT increased with age in men except at the nasion and midphiltrum. Suzuki on Japanese adult [29] stated that FSTT increases with age and more significantly at the menton. For this combined Nigerian ethnicities (CNME) most FSTT decreased with age though increased with age at the li, me and lm but these increases are minute. Also, unlike His report, the CNME mp decreased with age. And compared to the report by Suzuki, the menton showed insignificant changes with age.

#### Comparing with other populations

CNME midline value was compared with the FSTT of South African studies and with Rhine and Campbell (R & C) [16]. Rhine and Campbell was a study on black Americans in New Mexico. These findings are summarised in Table 11. Similarly, the CNME 18–32 (*A) data was compared with other studies with similar age brackets including A°, C & S and the Turkish 18–29 data as reported by Bulut et al., [5].

**Table 11:** Compares midline FSTT values with other populations (mm)

Data Table							Comparison Table						
FSTT points	CNME	Bulut (18 – 29)	A°	R & C	P & S	C & S	CNME – A°	CNME- R & C	CNME – P & S	CNME – C & S	*A - A°	*A – O	*A – C&S
Vertex	6.39	-	-	-	-	-	-	-	-	-	-	-	-
Supraglabella	4.56	4.04	5.21	4.75	5.36	4.7	0.65	−0.19	−0.80	−0.14	−0.36	0.81	0.15
Glabella	5.46	6.43	5.76	6.25	5.47	6.3	−0.3	−0.79	−0.01	−0.84	0.3	−0.37	−0.24
Nasion	6.09	7.23	7.03	6	4	6	0.94	0.09	2.09*	0.09	−0.4	−0.6	0.63
Mid-nasal	3.97	-	4.82	-	-	-	0.85	-	-	-	−0.45	-	-
Rhinion	3.35	2.96	3.08	3.75	2.88	2.7	0.27	−0.4	0.47	0.65	0.23	0.35	0.61
Midphiltrum	12.64	14.19	-	12.25	12.25	10.9	-	0.39	0.39	1.74*	-	−0.88	2.41*
Upper lip border	10.17	13.88	-	14	13.16	13.3	-	−3.83**	−2.99*	−3.13**	-	−2.96	−2.38*
Lower lip border	10.79	14.11	-	15	10.43	14.7	-	−4.21**	0.36	−3.91**	-	−3.67**	−4.26**
Labiomental	12.4	10.31	-	12	12.02	12.2	-	0.4	0.38	0.2	-	1.6	−0.29
Mental eminence	10.36	12.02	-	12.25	8.94	10.6	-	−1.89*	1.42	−0.24	-	−1.91*	−0.49
Menton	6.29	7.25	-	8	6.61	6.7	-	−1.71*	−0.32	−0.41	-	−0.92	−0.37

Aulsebrook et el.[34] = *A*°, Rhine and Campbell = R & C (black only data), Phillips and Smuts = P & S, Cavanagh and Steyn = C & S, CNME (18–32) = *A, Bulut et al., 2014 [5] = O (18–29). *= indicates significant level, **= more significant.

This Table 11 shows that CNME midline FSTT value is similar to values by Aulsebrook et al.[34] The biggest difference from the CNME and CNME 18–32 is at the n of 0.94 and 0.60 respectively. These differences are probably insignificant. Comparing CNME and CNME 18–32 with Rhine and Campbell showed more obvious differences at the ls, li, me and mn. With Phillips and Smuts there are differences at the n and ls and marginally at the mn. Compared with the only female South African study by Cavanagh and Steyn, showed that the Gauteng females had more FSTT values at most points except at the mid-philtrum. Contrary to this a lot of intra-population study [5, 8, 24, 42] have reported bigger FSTT values in males than females at most FSTT points. Although Shehata et al. [48], El-Mehallawi and Soliman [43] had both reported more FSTT in Egyptian female than their male. Comparing CNME 18–32 (*A) data with Turkish population data there were more robust differences in soft tissues. The Turkish young adult had more FSTT on most of the lower face at the ls, li, mn and the me except for the lm where the CNME (18–32) displaced more tissues. This study is similar to report by Stephan and Simpson [2] that FSTT values do not widely differ in different populations however, these males’ midline FSTT values among Nigeria, South Africa and Türkiye though similar are with evident differences at the lower 3rd of face. These differences may improve FFR and recognition among Nigerian adult males.

### Analysis of bilateral data

#### Effect of age

The CNME bilateral FSTT values displayed reduction with age increase, this was especially obvious in the older age, 63–100. The FSTT with the highest value was constantly the mid-masseter and lowest was persistently at the mid-lateral orbit. Most of these bilateral FSTT significantly showed more correlation with age increase and especially at 9 points (sm2, mm, im2, ol, go, lmx, sc, lno and ln). The fe showed the least relationship with age increase. These findings are similar to other published reports on men by Czekanowski.

#### Comparing with other populations

**Fig. 5:**
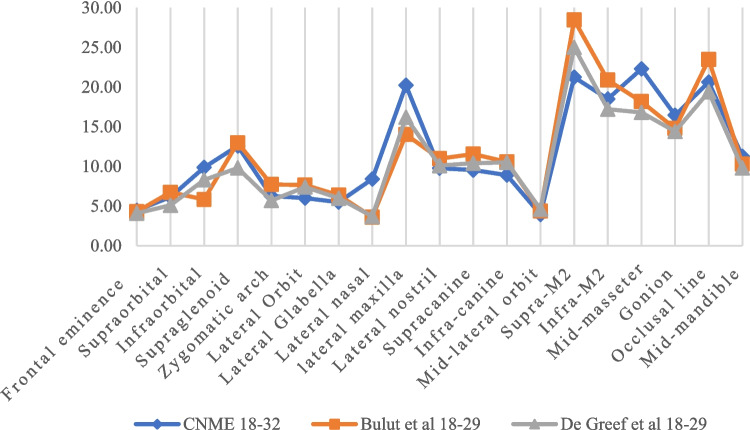
Comparing bilateral FSTT by different populations in early adulthood (mm)

**Fig. 6:**
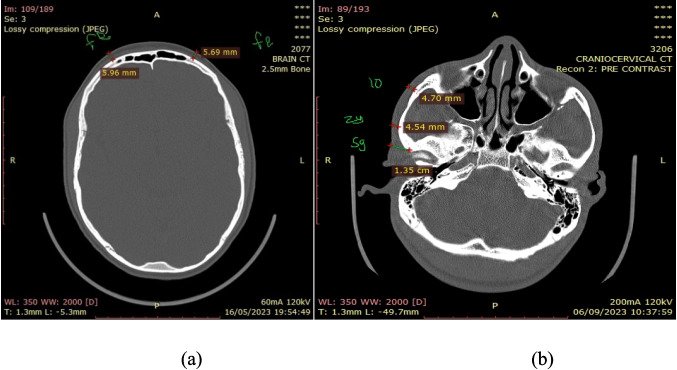
Measurement of the (**a**) frontal eminence (**b**) supraglenoid, zygomatic arch, lateral orbit

**Fig. 7:**
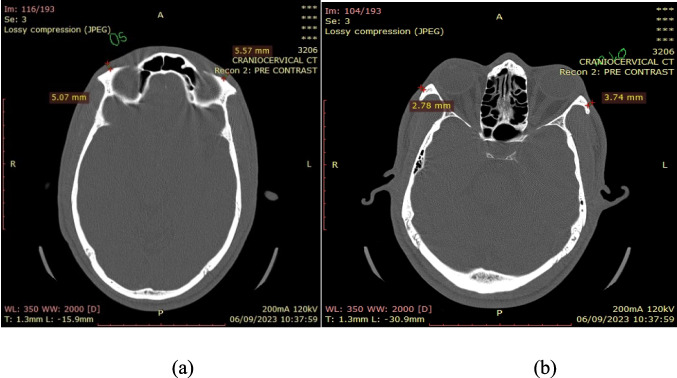
Measurement of the (**a**) supraorbital (**b**) mid-lateral orbit

His [7] study on adult male indicated that FSTTs increased with increased age except at the go. In this study the go increased in early life, with decrease in the 63–100. Manhein [53] on black American stated that FSTT decreased at the sc, go and sg. This is similar, amongst the CNME, sc decreased from 33–47 with a wider reduction of 2.33mm at the 63–100, the sg also decreased. Bulut et al., [5] similarly reported decrease in the sc by at least 2mm in the Türkiye adult males. Suzuki [29] study on Japanese men, stated that the masseter muscle decreased between the age at 60 to 80 years, likewise for the CNME the mm increased in the 33–47 but decreased in the 48–62 and with even more pronounce reduction in the 63–100 of 4.1mm, Table 9. Furthermore, this data was compared with other population studies as illustrated in Tables 12 and 13 and Fig. 5 as below. These Tables illustrates that the CNME adult males showed significantly more FSTT values than the Zulus (Aulsebrook et al.[34]) at oi (9.44, 6.56), sg (12.55, 5.91), ln (7.43, 4.8), mm (21.54, 18.05) respectively. CNME also showed more values than R & C at the oi, lm and the go. R & C had significantly elevated values at the fe, zy, lo and sm2, this may be because R & C was a cadaveric study. CNME compared with the P & S study showed that the Nigerian adult male had more FSTT at the oi, sg, sm2, im2 and go. CNME will equally have slightly more FSTT at the lo (6.06, 7.54). However, CNME compared with C & S showed that South African females had more FSTT at zy, sm2, im2, ol. CNME had more value only at the oi.

**Table 12:** Bilateral FSTT values for different population and comparison with CNME FSTT (mm)

Data Table							Comparison Table				
FSTT	CNME	A°	R&C	P&S	C&S	Hwang et al	A – CNME	(R & C) – CNME	(P & S) – CNME	(C & S) - CNME	Hwang et al - CNME
Frontal eminence	4.77	4.79	8.75	4.51	4.8	6.2	0.02	3.98*	−0.26	0.03	1.43
Supraorbital	5.73	6.05	4.75	5.46	6.8	7.2	0.32	−0.98	−0.27	1.07	1.47
Infraorbital	9.44	6.56	7.75	5.97	6.9	7.4	−2.88*	−1.69	−3.47**	−2.54*	−2.04*
Supraglenoid	12.55	5.91	11.75	9.1	12	12.6	−6.64***	−0.8	−3.45**	−0.55	0.05
Zygomatic arch	6.26	7.02	8.5	6.49	8.4	8.1	0.76	2.24*	0.23	2.14*	1.84
Lateral Orbit	6.06	-	13.25	7.54	-	8.6	-	7.19***	1.48	-	2.54*
Lateral Glabella	4.92	5.53	-	-	-	9.2	0.61	-	-	-	4.28**
Lateral nasal	7.43	4.8	-	-	-	7.3	−2.63*	-	-	-	−0.13
Lateral maxilla	20.09	-	17	-	-	18.6	-	−3.09**	-	-	−1.49
Lateral nostril	9.33	-	-	-	-	14.3	-	-	-	-	4.97**
Supracanine	8.8	-	-	-	-	11	-	-	-	-	2.2*
Infra-canine	8.42	-	-	-	-	12.2	-	-	-	-	3.78*
Mid-lateral orbit	3.61	-	-	-	-	4.8	-	-	-	-	1.19
Supra-M2	19.93	-	22	12.68	30.1	28.5	-	2.07*	−7.25***	10.17***	8.57***
Infra-M2	17.34	-	16.5	13.13	21.7	21.1	-	−0.84	−4.21*	4.36**	3.76**
Mid-masseter	21.54	18.05	-	-	22.4	19.5	−3.49**	-	-	0.86	−2.04*
Gonion	16.57	-	14.75	14.2	17.9	14.3	-	−1.82*	−2.37*	1.33	−2.27*
Occlusal line	19.51	-	19	19.06	21.7	22.9	-	-	−0.45	2.19*	3.39**
Mid-mandible	11.71	-	-	-	-	7.9	-	-	-	-	−3.81**

*= indicates significant level, **= more significant

**Table 13:** Differences between the CNME 18–32 (CNME A) bilateralFSTT values and other populations (mm)

FSTT	CNME 18–32	Bulut et al., 18–29	De Greef et al. 18–29	CNME A - Bulut et al.,	CNME A - De Greef
Frontal eminence	4.46	4.3	4.1	0.16	0.36
Supraorbital	6.14	6.74	5.1	−0.60	1.04
Infraorbital	9.87	5.83	8.3	4.04**	1.57*
Supraglenoid	12.58	12.98	9.8	−0.40	2.78*
Zygomatic arch	6.27	7.73	5.7	−1.46	0.57
Lateral Orbit	6.01	7.65	7.4	−1.64*	−1.39
Lateral Glabella	5.50	6.38	6	−0.88	−0.50
Lateral nasal	8.41	3.61	3.7	4.80***	4.71***
lateral maxilla	20.23	14.02	16.2	6.21***	4.03**
Lateral nostril	9.77	10.98	10.1	−1.21	−0.33
Supracanine	9.54	11.55	10.4	−2.01*	−0.86
Infra-canine	8.91	10.58	10.5	−1.67*	−1.59*
Mid-lateral orbit	3.94	4.37	4.6	−0.43	−0.66
Supra-M2	21.24	28.46	25	−7.22***	−3.76**
Infra-M2	18.50	20.87	17.2	−2.37*	1.30
Mid-masseter	22.49	18.17	16.8	4.12**	5.49***
Gonion	16.45	14.78	14.4	1.67*	2.05*
Occlusal line	20.63	23.48	19.4	−2.85**	1.23
Mid-mandible	11.27	10.3	9.8	0.97	1.47

*= indicates significant level, **= more significant

The CNME and CNME 18–32 (CNME A) were also compared with other population studies including the Hwang et al., [42] and similar age brackets with Bulut et al., (Turkish study) [5] and De Greef et al. (Belgian study) [11], Tables 12 and 13. CNME compared with Hwang et al. showed greater FSTT thickness values than the Hwang et al. at the oi, lm, mm, go, mdm but significantly less values than the Korean males at the fe, os, zy, lo, lg, lno, sc, ic, sm2, im2 and ol. CNME 18–32 compared with the Turkish 18–29 showed more FSTTs values at the oi, ln, lm, mm. Also, CNME 18–32 displayed more FSTT values than the De Greef et al. at the sg, ln, lm, mm. De Greef et al., (18–29) however showed more FSTT at only the lo, sm2, ic and slightly more FSTT at the sc (9.25, 10.4 respectively), difference of 1.15mm.

Hence, when compared with other populations values these CNME data of the right side of the face shows more variations than midline data. As previously stated by other studies apposite average population specific FSTT are important for precision of FFR [5, 38, 42, 58], having at least all 10 midline points (sg, g, n, ns or rh, mp, ls, li, lm, me, mn) and the most number of bilateral FSTT points should enhance FFR. These study demonstrates that there are variations between the right side of the face in the male adult CNME and other populations.

## Limitations and future work

Although in this project we had a good number of FSTT points however we struggled to recruit subjects especially for the older age brackets especially in the 63 to 100. A CT scanner of lesser thickness (0.5mm) would have more sensitive than a 2.5mm used in this study. In future works we hope to investigate the FSTT values of older age brackets. An obvious limitation of this study is the very limited data and analysis on BMI, in future works this will hopefully be explored and with greater number of subjects.

## Conclusion

By using CT scans in this collaborative study, a database of FSTT for the combined Nigerian male ethnicities (CNME) was created and compared with the published FSTT values of adult males of South Africa, Türkiye, Belgium, Korea and with that of South African adult females. Differences in these FSTT have been discussed. The Nigerian, South African and black American males’ midline FSTT data are similar in the upper two-thirds of the face, however, there are obvious differences in the lower 3rd of the face (lips and chin region). Furthermore, the bilateral FSTTs when compared shows even wider variations. The increased variation in FSTT values at the sides of the face as compared to the midline are due to greater thicknesses of soft tissue and the wider areas of the maxilla and mandible in the bilateral areas. These differences further strengthen the fact that preferably ethnic specific FSTT values will be more applicable for FFR. Little provision was given to BMI in this study due to limited sample size and difficulties in obtaining scans remotely.

## Data Availability

We declare the original raw data are available and can be provided on request. Not published due to the large size of the datasheet.

## Appendix

See Figs. 6 and 7 here.
